# Oral microbiome components predict response to neoadjuvant chemotherapy in triple-negative breast cancer patients

**DOI:** 10.3389/fonc.2025.1546044

**Published:** 2025-07-23

**Authors:** Xiaoyan Fu, Ze Huang, Zongyan Li, Zuxiao Chen, Zhijie Wu, Qingyu Yang, Haiyan Li

**Affiliations:** ^1^ Department of General Surgery (Breast Surgery), The Sixth Affiliated Hospital, Sun Yat-sen University, Guangzhou, China; ^2^ Biomedical Innovation Center, The Sixth Affiliated Hospital, Sun Yat-sen University, Guangzhou, China

**Keywords:** oral microbiome, triple-negative breast cancer, neoadjuvant chemotherapy sensitivity, response-prediction model, biomarker

## Abstract

The oral microbiome has emerged as a critical biomarker and regulator in cancer development and treatment response, garnering increasing attention from researchers. However, its specific role in breast cancer, particularly in triple-negative breast cancer (TNBC), remains poorly understood. The influence of the oral microbiome on chemotherapy sensitivity in TNBC, along with the underlying molecular mechanisms, remains unclear. Further investigation is needed to assess its potential as a biomarker for predicting chemotherapy sensitivity in this patient population. In the present study, significant differences in the composition of the oral microbiome were observed among patients with varying chemotherapy sensitivities for TNBC patients. Additionally, notable changes in the oral microbiome were noted after chemotherapy in patients with favorable responses to treatment. Our analysis revealed that chemotherapy-sensitive patients had higher levels of *Lactobacillus* and *Neisseria* species, alongside lower levels of *Clostridium* species. Post-chemotherapy, patients with positive responses demonstrated an increase in *Clostridium* and *Microbacterium* species, along with a decrease in *Streptococcus* and *Neisseria*. In contrast, no significant changes were observed in the microbiota of patients with poor chemotherapy responses. A classifier based on these microbial biomarkers yielded an area under the curve (AUC) value of 77.3% (95% CI: 60.5%-94.2%), supporting the potential of the oral microbiome as a predictive tool for chemotherapy sensitivity in TNBC. Given its simplicity, non-invasiveness, and repeatability, the oral microbiome holds promise as a valuable biomarker for predicting neoadjuvant chemotherapy sensitivity in TNBC patients.

## 1 Introduction

Although advancements in neoadjuvant chemotherapy have enhanced the pathological complete response (pCR) rates in triple-negative breast cancer (TNBC) patients (1), a subset remains unresponsive due to low treatment sensitivity. Consequently, predicting the efficacy of chemotherapy regimens has become a critical challenge in clinical practice to facilitate the timely selection of optimal therapeutic strategies for TNBC.

The human microbiome represents a complex and dynamic ecosystem that extends beyond the gut, encompassing distinct microbial communities across various body sites. Among these, the oral microbiome has garnered increasing attention—not only for its pivotal role in maintaining local oral health and contributing to disease pathogenesis, but also as a key component of the broader human microbiome network. Under normal conditions, a mutual equilibrium is maintained between the host and the oral microbiome. However, external environmental factors, such as pH, temperature fluctuations, or antibiotic exposure, can disrupt this balance by altering the composition of the resident microbiota, potentially leading to oral or systemic diseases. Importantly, the oral microbiome does not exist in isolation. Beyond its prolonged colonization within the oral cavity, oral-derived bacteria can translocate to other regions of the body via the digestive system or bloodstream, contributing to infections and localized inflammation (2, 3). Compelling evidence suggests that such bacterial migration extends to the mammary gland, contributing to the establishment of a resident mammary microbiota. Research has demonstrated that a substantial proportion of bacteria found in the oral and gastrointestinal microbiomes are also present in breast milk and breast tissue. These microorganisms may reach the mammary gland through several potential pathways: (a) infiltration through the skin and nipple; (b) translocation and colonization via the digestive and reproductive tracts; and (c) migration through the blood and lymphatic systems to the breast lobules and ducts (4–7). This provides evidence of a direct link between the oral microbiome and the mammary microbiome.

The existence of a mammary microbiome suggests a potential role in regulating breast physiology and contributing to the development of breast pathology. Human microbiota, particularly the gut microbiome, has been shown to influence the development of breast cancer through mechanisms such as estrogen metabolism, modulation of inflammatory responses, and immune regulation (8). Moreover, specific characteristics of the gut microbiome have been found to correlate with distinct breast cancer subtypes, potentially aiding in the prediction of treatment responses and patient prognosis (8, 9). However, the potential impact of the mammary microbiome—potentially seeded in part by oral bacteria—on breast cancer biology and therapeutic response remains an emerging area of research. Building on the previously established association, recent studies have revealed a potential direct link between breast cancer and the oral microbiome. Evidence suggests a significant association between periodontal disease and an increased risk of breast cancer, with women affected by periodontal disease being more susceptible (10). Periodontal disease, caused by specific bacterial species, notably *Fusobacterium nucleatum*, may facilitate hematogenous dissemination and colonization at breast cancer sites. This process can suppress anti-tumor immunity and promote the progression of breast cancer (11).

Consequently, the oral microbiome has emerged as a compelling focal point of research, serving both as a potential source and a biomarker of microbial populations that may shape the mammary microenvironment. It is implicated in the initiation, progression, and treatment response of breast cancer. However, its influence on chemotherapy sensitivity in TNBC and the underlying molecular mechanisms remain inadequately understood. The objective of this study was to apply the conceptual framework of the oral microbiota as a window into mammary microbiota influence to examine the alterations in the oral microbiome of TNBC patients before and after chemotherapy, and to assess the potential of the oral microbiome as a non-invasive biomarker for predicting chemotherapy sensitivity in TNBC.

## 2 Materials and methods

### 2.1 Study design and study participants

**Figure 1:**
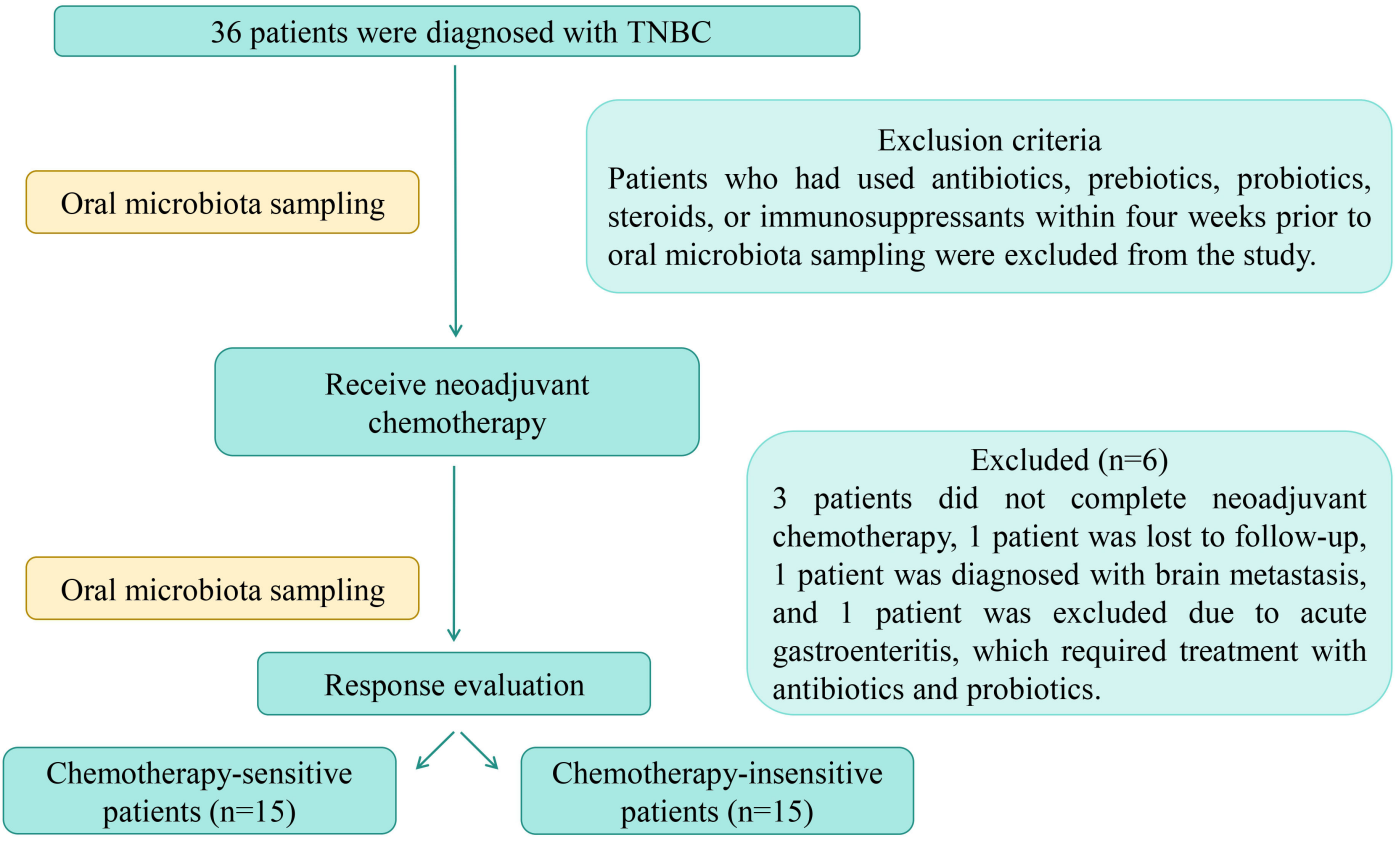
Flow diagram of inclusion and exclusion criteria.

We enrolled 36 patients with TNBC who received neoadjuvant chemotherapy between April 2021 and February 2023. Patients who had used antibiotics, prebiotics, probiotics, steroids, or immunosuppressants within four weeks prior to oral microbiota sampling were excluded from the study. These 36 patients were diagnosed with triple-negative breast cancer (TNBC) based on pathological confirmation prior to treatment and fulfilled at least one of the following criteria: clinically positive lymph nodes or tumors measuring 2 cm or larger, with no evidence of distant metastasis. All participants received a 21-week treatment regimen consisting of epirubicin and cyclophosphamide, followed by paclitaxel, for a total of eight cycles. During this period, three patients did not complete neoadjuvant chemotherapy, one patient was lost to follow-up, one was diagnosed with brain metastasis during neoadjuvant therapy, and one patient was excluded due to acute gastroenteritis, which required treatment with antibiotics and probiotics (**Figure 1**).

Post-neoadjuvant chemotherapy, pathological evaluation was performed using the Miller-Payne grading system. G1-G2 was classified as chemotherapy-insensitive with poor response, while G3-G5 was categorized as chemotherapy-sensitive with a favorable response. Ultimately, the study included 15 chemotherapy-sensitive patients and 15 chemotherapy-insensitive patients.

### 2.2 Oral microbiome sampling and DNA extraction

Oral microbiome samples were collected from all patients using oral swabs both before and after chemotherapy. Sampling was performed in the morning under fasting conditions, with patients refraining from food intake and oral hygiene for at least 12 hours prior to collection. A sterile cotton swab moistened with normal saline was used to gently swab the buccal mucosa, upper jaw, teeth, and posterior tongue to collect microbial specimens. After swabbing, the samples were immediately placed into sterile cryotubes. For each sample, 2–3 swabs were collected, the tube was securely sealed, and the specimens were promptly transferred into an insulated container with crushed ice or ice packs. Samples were then transported to the laboratory for immediate DNA extraction or stored at -80°C for no more than 4 hours if short-term storage was necessary. DNA was extracted using the cetyltrimethylammonium bromide (CTAB) method, following the manufacturer’s instructions. This reagent is specifically optimized for isolating DNA from minimal amounts of biological material and has demonstrated efficacy in extracting DNA from a wide variety of bacterial species. Nuclease-free water was used as a blank control. The total DNA was eluted in 50 μL of elution buffer and stored at -80°C until polymerase chain reaction (PCR) analysis, which was conducted by LC-Bio Technology Co., Ltd., Hangzhou, Zhejiang Province, China (12–15).

### 2.3 Data analysis

The samples were sequenced on an Illumina NovaSeq platform in accordance with the manufacturer’s instructions, as provided by LC-Bio. Paired-end reads were assigned to the samples based on their unique barcodes and truncated by cutting off the barcode and primer sequences. Paired-end reads were merged using the FLASH software. The quality of the raw reads was assessed and filtered according to specific criteria in order to obtain high-quality clean tags, as outlined in the fqtrim (v0.94) protocol. Chimeric sequences were filtered using the Vsearch software (v2.3.4). Following dereplication using DADA2, feature tables and feature sequences were obtained, and alpha and beta diversity were calculated by normalizing to the same sequences randomly. Subsequently, the feature abundance was normalized according to the relative abundance of each sample using the SILVA (release 138) classifier. Alpha diversity was employed to assess the diversity of species within a sample, utilizing five indices: Chao1, Observed species, Goods coverage, Shannon, and Simpson. These indices were calculated for all samples using the QIIME2 software. Beta diversity was calculated using QIIME2, and visualizations were generated using R packages. Beta diversity assesses inter-sample or inter-group variability by computing distance metrics, with the weighted UniFrac method used in this study. Sequence alignment was performed using BLAST, and feature sequences were annotated using the SILVA database for each representative sequence. All data analyses were conducted on the R statistical computing platform. Microbial community shifts during disease progression at both the phylum and genus levels were visualized using bubble plots and heatmaps, generated with the pheatmap, stats, ggplot2 (v3.2.0), and corrplot packages in R. Linear Discriminant Analysis Effect Size (LEfSe) was used to identify differential features among groups by integrating non-parametric statistical tests, linear discriminant analysis (LDA), and effect size estimation. Specifically, the Kruskal-Wallis test was used to detect features with significant differences across groups, followed by LDA to estimate their relative impact. Significance analysis was performed for all taxa at each taxonomic level. Among the taxa with *p* < 0.05, the top 20 most abundant were selected for bar plot visualization. Other diagrams were created with the R package (v3.5.2) (12–14). The performance of the classifier was assessed using the area under the receiver operating characteristic (ROC) curve. ROC curve analysis was conducted using SPSS (v25.0).

## 3 Results

### 3.1 Distinct variations in the oral microbiome were observed among TNBC patients with differing responses to neoadjuvant chemotherapy

To investigate the composition of the oral microbiome in TNBC patients, microbiome samples were collected from 30 patients undergoing neoadjuvant chemotherapy, both before and after treatment. The samples were subjected to 16S rDNA sequencing for microbiome profiling. Patients were stratified into two groups based on their response to chemotherapy: the sensitive group, consisting of 15 patients with a favorable response, and the insensitive group, comprising the remaining 15 patients. The baseline demographics and clinical characteristics of both groups are summarized in **Table 1**.

**Table 1:** Baseline characteristics of the TNBC patients enrolled in this study.

Baseline characteristics	TNBC patients (n=30)		*P*-value
	IS Group (n=15)	S Group (n=15)	
Age (year)			
< 65	10	15	0.042
≥ 65	5	0	
Clinical T stage			
cT1	2	0	0.445
cT2	7	10	
cT3	4	3	
cT4	2	2	
Clinical N stage			
cN+	12	14	0.283
cN0	3	1	
Tumor Grade			
G1	2	1	0.717
G2	8	10	
G3	5	4	
Ki-67			
< 20%	5	4	0.690
≥ 20%	10	11	

TNBC, triple-negative breast cancer; IS, insensitive; S, sensitive.

**Figure 2:**
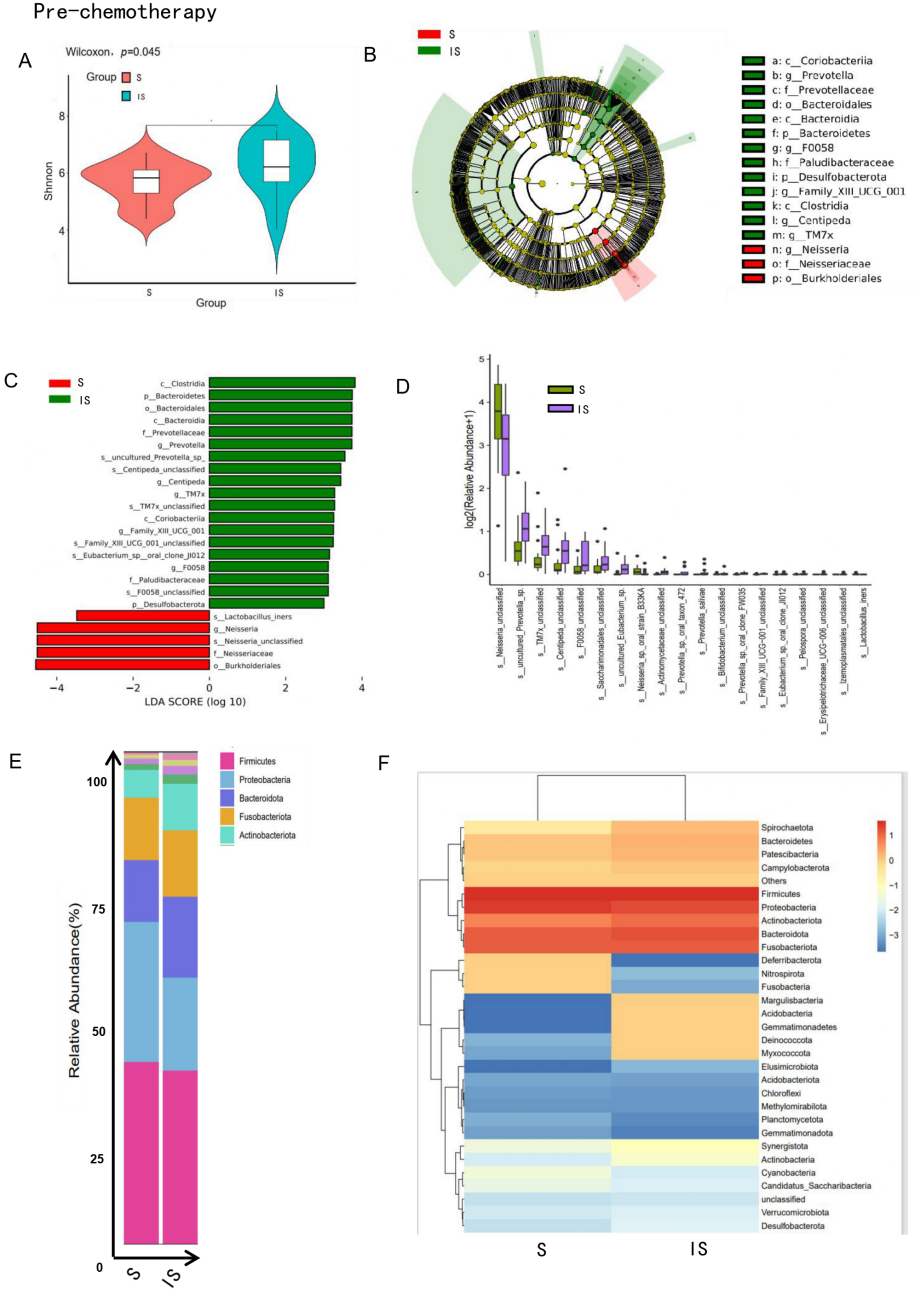
The oral microbiome of TNBC patients demonstrated notable distinctions based on their responses to neoadjuvant chemotherapy. **(A)** The violin plot illustrated the distribution of Shannon’s index for the bacterial community across each compartment. Significant differences in oral microbial α-diversity were observed between the chemotherapy-sensitive and non-sensitive groups, with the non-sensitive group exhibiting greater microbial richness (*p* < 0.05). **(B, C)** Differentially abundant taxa between chemotherapy-sensitive and -insensitive samples, identified via Linear Discriminant Analysis (LDA) Effect Size (LEfSe), were visualized using a cladogram **(B)** and histogram **(C)**. All listed taxa showed statistically significant enrichment in their respective groups (Kruskal–Wallis test, *P* < 0.05; LDA score > 2), with green indicating the insensitive group and red the sensitive group. LEfSe analysis demonstrated increased relative abundances of *Lactobacillus*, *Neisseria*, and *Burkholderiales* in the chemotherapy-sensitive group, whereas *Clostridia*, *Bacteroidetes*, *Prevotellaceae*, and *Coriobacteriia* were more prevalent in the chemotherapy-insensitive group. **(D)** The boxplot illustrated the relative abundances of differentially enriched taxa between the chemotherapy-sensitive (green) and -insensitive (purple) groups prior to treatment, as identified by LEfSe analysis (*P* < 0.05). Notably, *Neisseria* was significantly more abundant in the sensitive group, whereas *Prevotella*, *TM7X*, and *Centipeda* were enriched in the insensitive group. **(E)** Based on the species annotation results, select the top 15 strains in terms of maximum abundance at the phylum level in each group to generate a stacked bar chart of relative species abundance. The oral microbiome of both chemotherapy-sensitive and insensitive groups exhibited a similar predominance in their overall composition. **(F)** According to the relative abundance table of species, the community composition data of the top 30 in terms of relative abundance at each classification level are clustered based on the abundance distribution of classification units or the similarity degree among samples. The classification units and samples are sorted respectively according to the clustering results. *Nitrospirota*, *Fusobacteria*, *Margulisbacteria* and *Acidobacteria* were more abundant in the chemotherapy-sensitive group; *Gemmatimonadetes*, *Deinococcota* and *Myxococcota* were enriched in the chemotherapy-insensitive group. The scale bar (−3 to 1) represents the log-fold change in bacterial abundance, indicating increases or decreases in relative abundance.

The analysis revealed significant differences in α diversity between the two groups (p < 0.05), the α-diversity was higher in the chemotherapy-insensitive cohort (**Figure 2A**). High-dimensional comparison using LEfSe identified distinct microbial profiles associated with chemotherapy response. Specifically, the chemotherapy-sensitive group exhibited higher proportions of *Lactobacillus*, *Neisseria*, and *Burkholderiales*, while *Clostridia*, *Bacteroidetes*, *Prevotellaceae*, and *Coriobacteriia* were more prevalent in the chemotherapy-insensitive cohort (**Figures 2B, C**).

Taxonomic analysis revealed distinct differences in the oral microbiome composition between chemotherapy-sensitive and chemotherapy-insensitive TNBC patients. Specifically, *Neisseria* was more abundant in the oral microbiome of chemotherapy-sensitive patients, while *Prevotella*, *TM7X*, and *Centipeda* were enriched in those with chemotherapy insensitivity (**Figure 2D**). Across all samples, regardless of chemotherapy sensitivity, the dominant phyla included *Firmicutes*, *Proteobacteria*, *Bacteroidota*, *Fusobacteriota*, and *Actinobacteriota* (**Figure 2E**). However, significant differences were observed in the relative abundance of *Nitrospirota*, *Fusobacteria*, *Margulisbacteria*, and *Acidobacteria* between the two groups, with *Gemmatimonadetes*, *Deinococcota*, and *Myxococcota* being more prevalent in chemotherapy-insensitive patients (**Figure 2F**).

### 3.2 The composition of the oral microbiome in TNBC patients who responded to neoadjuvant chemotherapy exhibited notable changes following treatment

**Figure 3:**
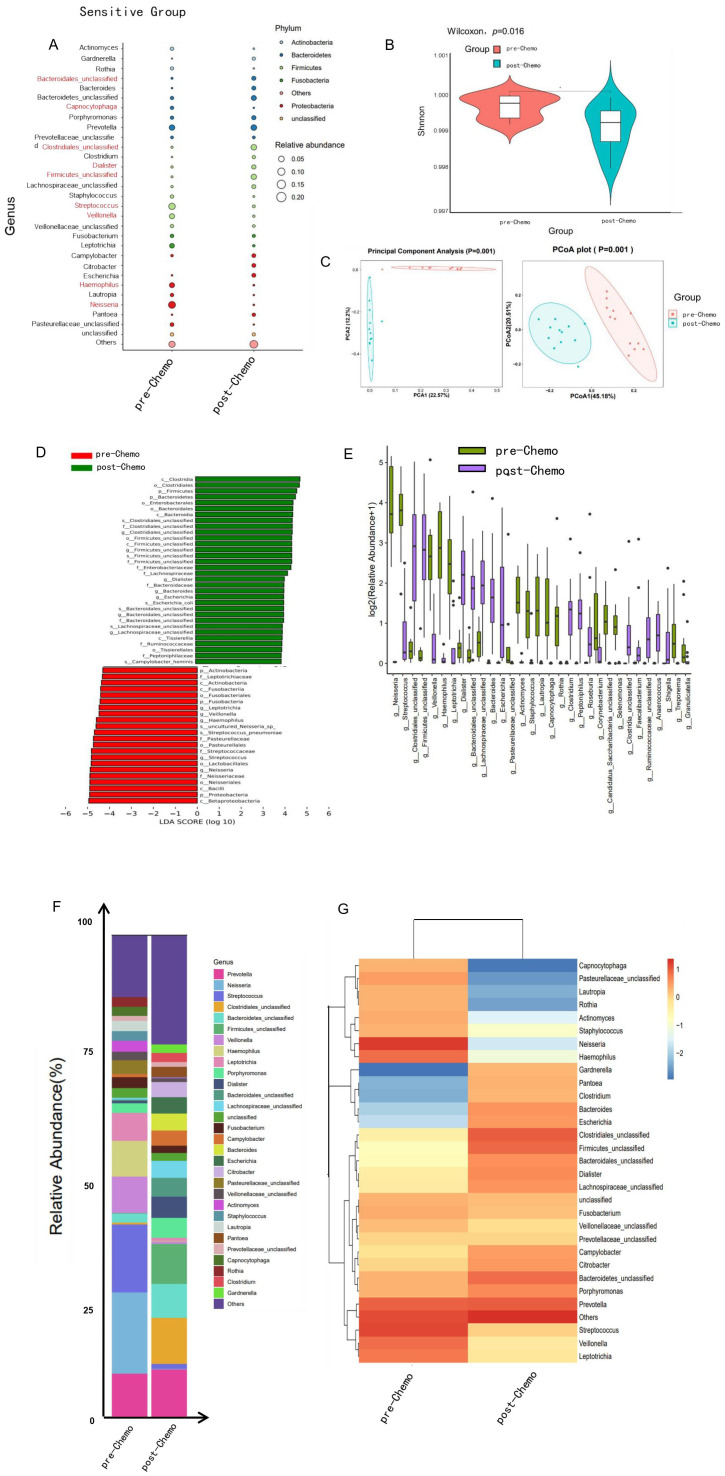
The composition of the oral microbiome in TNBC patients responsive to neoadjuvant chemotherapy exhibited notable alterations after chemotherapy. **(A)** The balloon plot revealed notable alterations at the genus level in patients responsive to neoadjuvant chemotherapy before and after treatment, both in terms of composition and relative abundance. **(B)** The violin plot showed the distribution of Shannon’s index of the bacterial community in each compartment. Prior to chemotherapy, α-diversity was significantly higher in chemotherapy-sensitive patients (*p* < 0.05), indicating greater microbial richness. **(C)** Principal Component Analysis (PCA) and Principal Coordinates Analysis (PCoA) revealed significant differences in oral microbiome composition before and after chemotherapy (PERMANOVA, *p* = 0.001, weighted UniFrac), along with a reduction in microbial diversity post-treatment. **(D)** High-dimensional analyses using LEfSe and taxonomic profiling further highlighted notable shifts in bacterial distribution within the chemotherapy-sensitive group after treatment. **(E)** The boxplot displayed the relative abundances of differentially abundant taxa in the sensitive group before (green) and after (purple) chemotherapy, as identified by LEfSe (*P < 0.05). **(F, G)** The stacked bar chart and heatmap showed significant alterations in the composition of oral microbiome at the genus level before and after chemotherapy.

In addition to profiling the distinct oral microbiota composition in patients with varying responses to chemotherapy, a detailed analysis was conducted to examine temporal changes in the oral microbiota throughout treatment. Sequential sampling of oral microbiota before and after chemotherapy facilitated the evaluation of microbial alterations during neoadjuvant chemotherapy. The analysis revealed notable shifts in microbiota diversity and relative abundance were observed at the phylum level, with significant increases in *Bacteroidales*, *Clostridiales*, and *Firmicutes* following chemotherapy relative to baseline levels (**Figure 3A**). Additionally, oral microbial α-diversity exhibited statistically significant differences between pre-chemotherapy and post-chemotherapy samples in sensitive patients (*p* < 0.05) (**Figure 3B**). β-diversity analysis further highlighted distinct patterns through Principal Component Analysis (PCA) and Principal Coordinate Analysis (PCoA), demonstrating reduced microbial diversity post-chemotherapy in this patient group (**Figure 3C**).

High-dimensional analysis using LEfSe, alongside taxonomic evaluation, identified notable changes in the distribution of bacterial taxa among patients receiving neoadjuvant chemotherapy. Prior to treatment, the oral microbiome was characterized by elevated levels of *Leptotrichiaceae*, *Neisseria*, *Streptococcus*, and *Bacillus*. Following chemotherapy, an increased abundance of *Clostridia*, *Bacteroidetes*, and *Firmicutes* was observed (**Figures 3D, E**). Furthermore, significant modifications in the oral microbiota composition were evident at the genus level between pre- and post-treatment samples (**Figures 3F, G**).

### 3.3 The oral microbiome composition in TNBC patients demonstrating insensitivity to neoadjuvant chemotherapy remained largely unchanged following treatment

**Figure 4:**
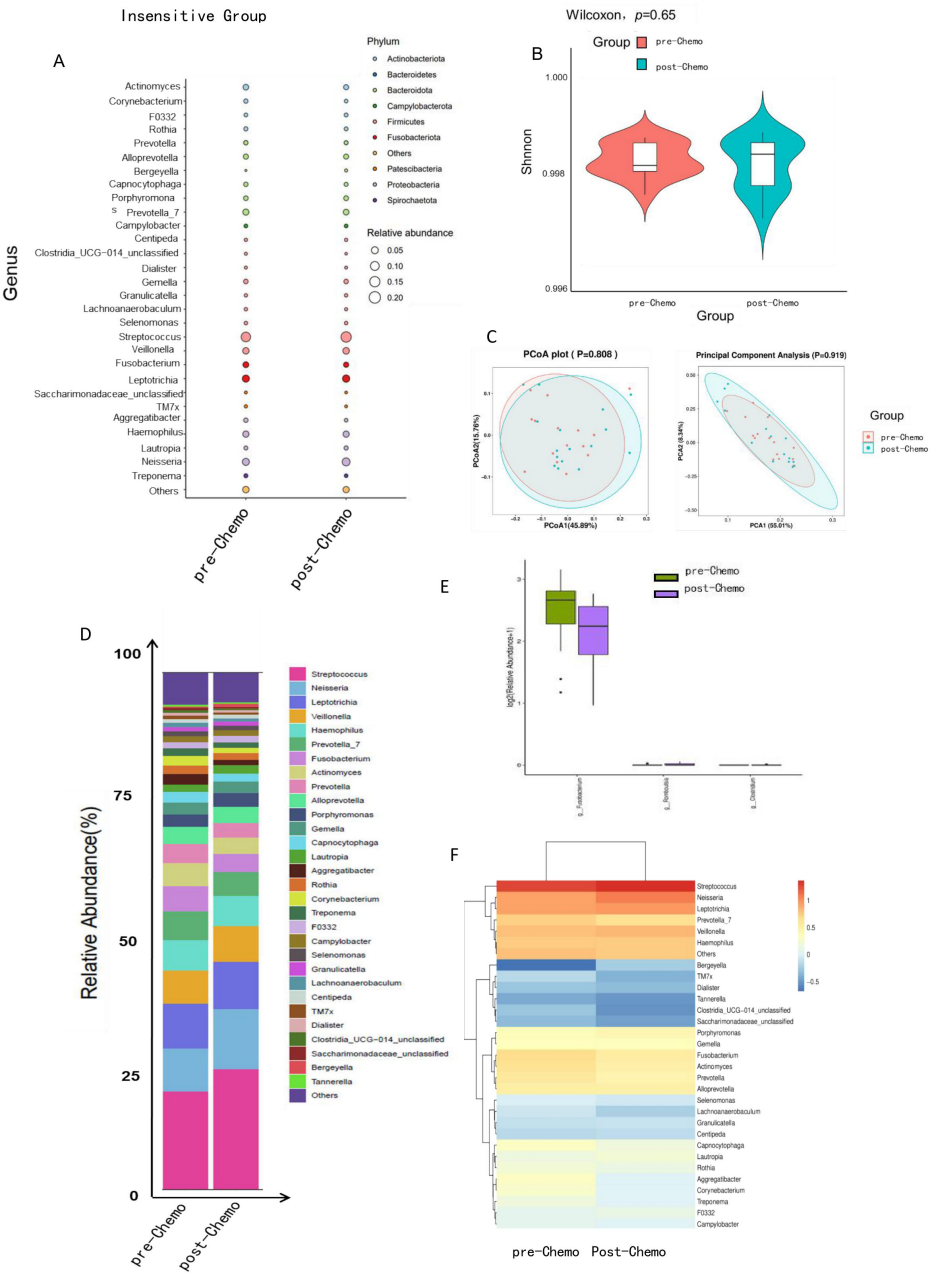
The composition of the oral microbiome in TNBC patients resistant to neoadjuvant chemotherapy remained relatively unchanged following treatment. **(A)** The balloon plot showed no significant differences in oral microbial abundance before and after chemotherapy. **(B)** The violin plot illustrated the distribution of Shannon’s index, indicating no significant change in oral microbial α-diversity within the chemotherapy-insensitive group (*p* > 0.5). **(C)** PCA and PCoA of β-diversity revealed no notable shifts in microbial community structure before and after treatment in the chemotherapy-insensitive group (PERMANOVA, *p* > 0.5, weighted UniFrac). **(D–F)** Taxonomic and bacterial composition analyses, as presented in the stacked bar chart, boxplot, and heatmap, further corroborated the absence of significant differences in the oral microbiome of chemotherapy-insensitive patients before and after chemotherapy.

No significant changes were observed in genus levels of the oral microbiome in patients who were insensitive to neoadjuvant chemotherapy before and after treatment. These findings suggest that chemotherapy response may be closely associated with the baseline composition of the oral microbiome. Analysis of oral microbial abundance (**Figure 4A**) and α-diversity (**Figure 4B**) demonstrated no significant differences before and after chemotherapy (*p* > 0.5). Similarly, β-diversity analysis showed no significant variation in microbial diversity pre- and post-treatment in the non-responsive group (**Figure 4C**). These observations were corroborated by taxonomic analysis and bacterial composition assessments (**Figures 4D–F**).

### 3.4 No significant association was observed between the composition of the oral microbiome and chemotherapy sensitivity in TNBC patients following neoadjuvant chemotherapy

**Figure 5:**
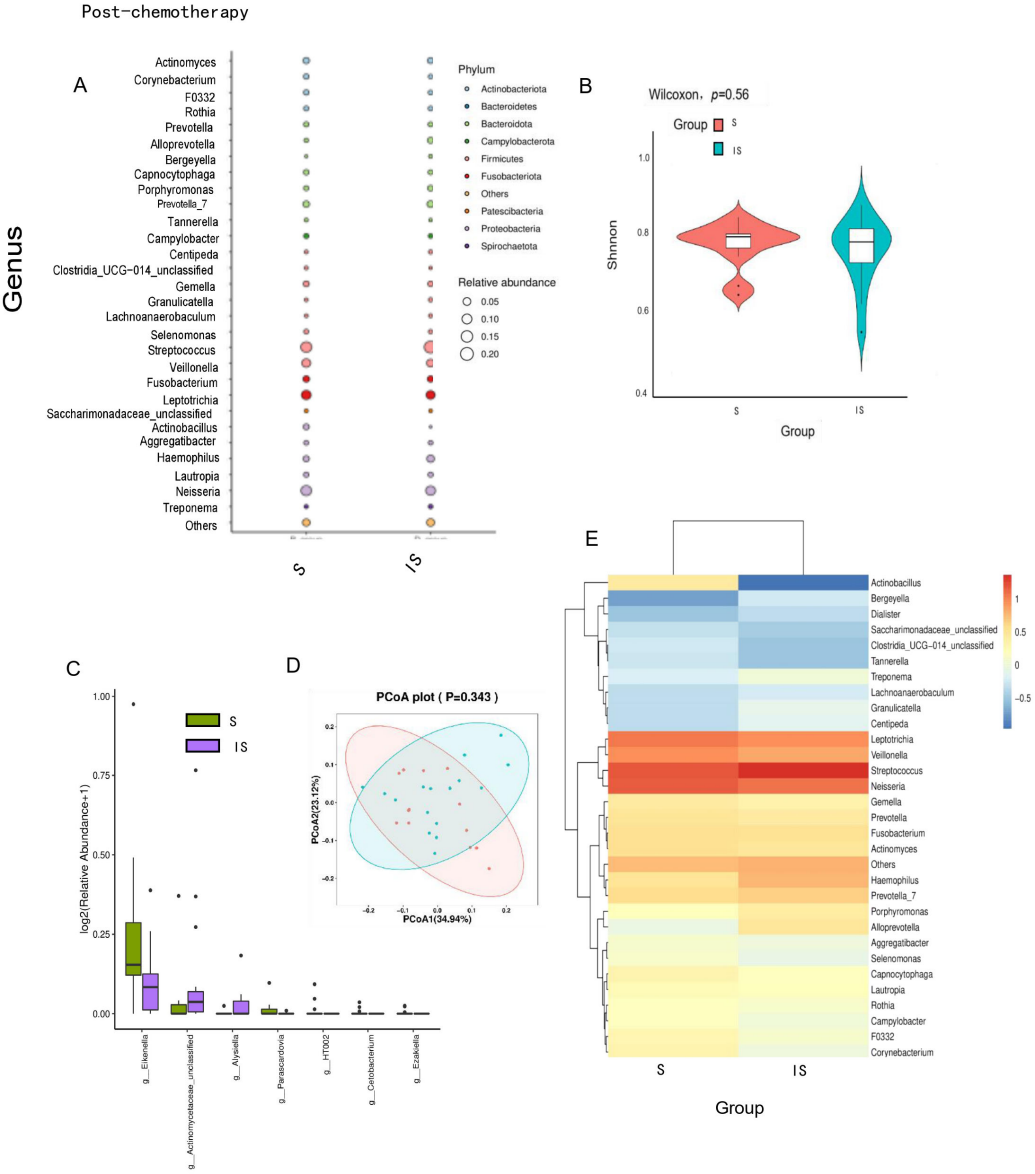
No significant association was observed between the composition of the oral microbiome and chemotherapy sensitivity in TNBC patients following neoadjuvant chemotherapy. **(A)** The balloon plot demonstrated that oral microbial abundance did not differ significantly following chemotherapy. **(B)** The violin plot indicated no significant change in oral microbial α-diversity after chemotherapy (*p* > 0.5). **(C)** Analysis of β diversity revealed no notable differences in microbial diversity before and after chemotherapy. **(D, E)** Taxonomic and bacterial composition analyses further corroborated the absence of significant alterations in the oral microbiome following chemotherapy.

A comparative analysis of the oral microbial composition in TNBC patients following neoadjuvant chemotherapy revealed no significant association between the oral microbiome and chemotherapy sensitivity (**Figure 5A**). There were no significant differences in oral microbial α diversity between groups (*p* > 0.5) (**Figure 5B**). Similarly, β diversity analysis demonstrated no significant variation in the oral microbiome post-chemotherapy, nor any correlation with initial chemotherapy sensitivity (**Figure 5C**). These findings were further corroborated by taxonomic and bacterial composition analyses, which did not identify any significant changes (**Figures 5D, E**). Collectively, these results suggest that neoadjuvant chemotherapy influences the composition of the oral microbiota in chemotherapy-sensitive patients.

### 3.5 The oral microbiome has the potential to serve as a predictive biomarker for the efficacy of neoadjuvant chemotherapy in TNBC

**Figure 6:**
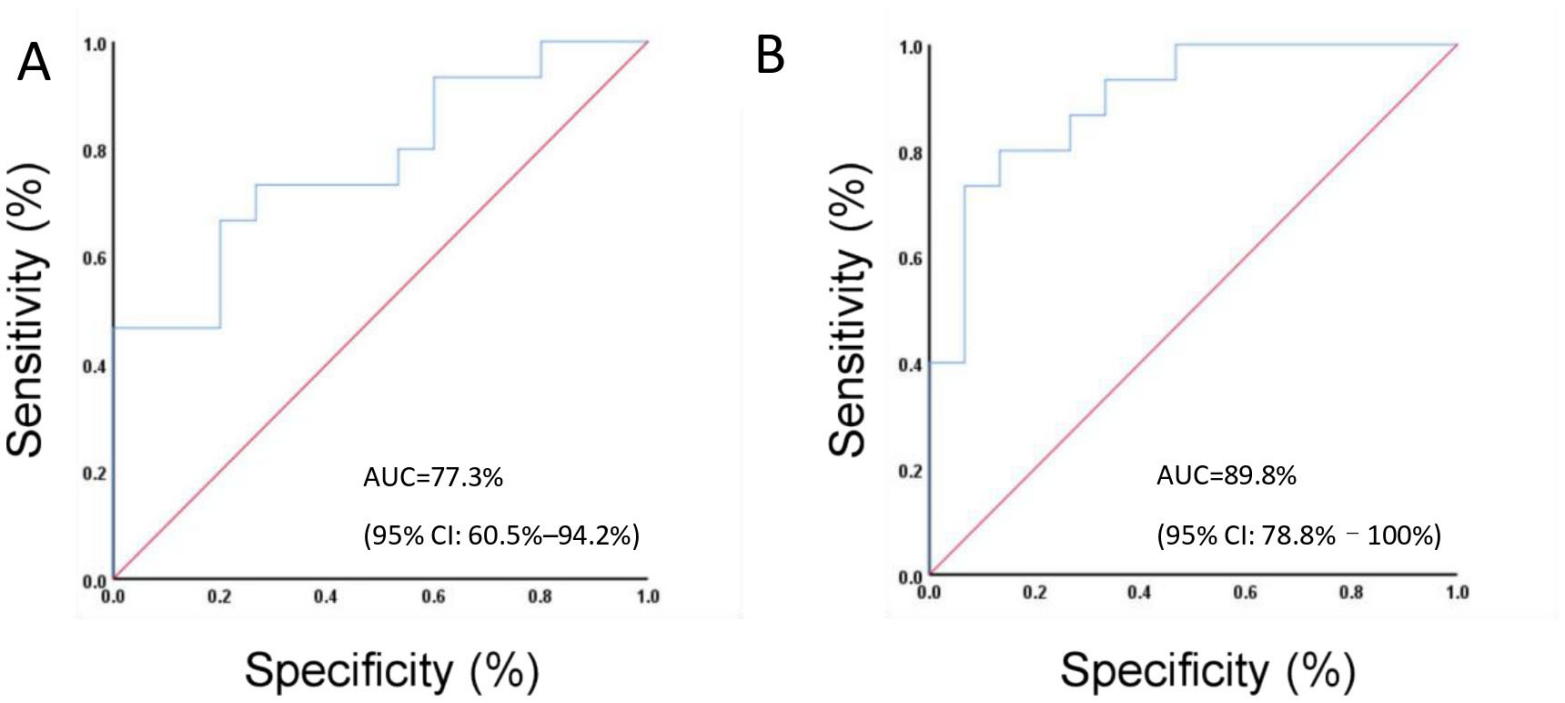
The oral microbiome shows potential as a predictive biomarker for the effectiveness of neoadjuvant chemotherapy in patients with TNBC. **(A)** Elevated levels of specific oral microbiome components in the chemotherapy-sensitive cohort were associated with an AUC of 77.3% (95% CI: 60.5%–94.2%). **(B)** Distinct oral microbiome components that were enriched in the chemotherapy-insensitive cohort exhibited an AUC of 89.8% (95% CI: 78.8%–100%).

To facilitate the clinical translation of our findings, we developed a predictive model for treatment response in locally advanced TNBC. A total of 27 potential discriminatory genera were identified based on the previously described taxonomic comparison analysis. These genera were subsequently used as input features for classification. Among these, oral microbiota components with elevated levels in the chemotherapy-sensitive cohort, such as *Neisseria* and *Streptococcus*, were selected for predicting therapeutic response, achieving an AUC of 77.3% (95% CI: 60.5%–94.2%) (**Figure 6A**). Similarly, microbiota components enriched in the chemotherapy-insensitive cohort, including *Firmicutes* and *Clostridiales*, were also incorporated into the model, yielding an AUC of 89.8% (95% CI: 78.8%–100%) (**Figure 6B**). These findings suggest that the oral microbiota may serve as a predictive biomarker for chemotherapy sensitivity in locally advanced TNBC, offering a simple, non-invasive, and reproducible approach for predicting neoadjuvant chemotherapy efficacy in breast cancer.

## 4 Discussion

This study introduces the potential of utilizing oral microbiome as a straightforward, reproducible, and noninvasive approach for predicting sensitivity to neoadjuvant chemotherapy in TNBC patients. This could uncover innovative biomarkers and therapeutic targets, offering valuable insights for the diagnosis and treatment of TNBC.

Disruptions in the ecological balance between the host and the microbiome can contribute to the development of diseases. Notably, the role of the microbiome in cancer has been widely studied and documented. While certain microbes have the capacity to directly initiate cancer through mechanisms like genotoxin-mediated mutagenesis, many others influence cancer progression indirectly by modulating the host immune system (16). The oral microbiome, recognized as the second-largest symbiotic microbial community within the human body and among its most diverse ecosystems, is closely associated with cancer progression (17). *Fusobacterium*, one of the most extensively studied oral microorganisms, has been implicated in cancer development through various mechanisms. These include inhibiting apoptosis, activating cell proliferation, promoting cellular invasion, inducing chronic inflammation, and directly producing carcinogenic substances (18). The oral microbiome possesses the ability to colonize the gastrointestinal tract via the circulatory system (19). Studies have shown that *Fusobacterium* is enriched in colorectal cancer (CRC) tissue, where it promotes tumorigenesis by creating a tumorigenic inflammatory environment conducive to tumor development while suppressing anti-tumor immune responses through both genetic and epigenetic mechanisms (20–23). Moreover, it has been associated with the enhancement of chemotherapy resistance in colorectal cancer. Additionally, it shows promise as a biomarker for the early detection of CRC as well as for prognostic and predictive applications (24–29).

However, studies investigating the oral microbiome in non-gastrointestinal solid tumors are relatively limited, particularly regarding its association with breast cancer, which is still not well understood. This study represents the first attempt to investigate the relationship between the oral microbiome and neoadjuvant chemotherapy responsiveness in patients with TNBC. This study identified higher relative abundances of *Lactobacillus*, *Neisseria*, and *Burkholderiales* in the chemotherapy-sensitive group, whereas *Clostridia*, *Bacteroidetes*, *Prevotellaceae*, and *Coriobacteriia* were more prominent in the chemotherapy-insensitive cohort. Additionally, significant compositional changes in the oral microbiome were observed in the chemotherapy-sensitive group following treatment, in contrast to the minimal alterations detected in the chemotherapy-insensitive group.

This study performed a comparative analysis of the oral microbiome in TNBC patients with differing levels of chemotherapy sensitivity prior to treatment, uncovering significant variations that point to a potential role of the oral microbiome in influencing neoadjuvant chemotherapy response. Additionally, post-chemotherapy alterations in the oral microbiome were predominantly observed in patients demonstrating chemosensitivity, suggesting that modulating the composition of the oral microbiome could impact the effectiveness of neoadjuvant chemotherapy in TNBC. Previous study has reported that concurrent antibiotic use during neoadjuvant pembrolizumab therapy in HER2-negative early-stage breast cancer is associated with increased residual cancer burden, suggesting a potential adverse effect of microbial dysbiosis (30). In the present study, chemotherapy-sensitive patients exhibited a higher relative abundance of *Lactobacillus* in their oral microbiome, a genus frequently found in probiotics and yogurt. Consequently, further investigation is warranted to explore whether augmenting these bacteria within the microbiome could enhance chemotherapy sensitivity in TNBC.

Our study further demonstrated that chemotherapy-sensitive patients exhibited a high abundance of *Neisseria* in their oral microbiome, which showed potential as a predictor of chemotherapy efficacy. However, current knowledge predominantly characterizes *Neisseria* as a pathogenic genus, including species such as *Neisseria meningitidis* and *Neisseria gonorrhoeae*. The role of *Neisseria* in tumor biology remains poorly understood, though some evidence suggests it may promote the initiation and progression of certain cancers, such as ovarian cancer, with studies indicating that vaginal infections (e.g., *Neisseria gonorrhoeae* or *Chlamydia trachomatis*) are associated with an increased risk of developing ovarian cancer (31). These findings appear contradictory to our results; however, research indicates that both pathogenic and non-pathogenic *Neisseria* species possess mechanisms to inhibit competing microbial populations, potentially antagonizing the growth of other microbial species (32). The possible inhibitory effects of *Neisseria* on components of the oral microbiome that might impair chemotherapy response remain unclear, highlighting an area of interest for further research.

The limitations of our study were twofold. Firstly, the study population was confined to a single institution, which may limit the generalizability of the findings. Expanding the cohort to include patients from both our institution and external centers is essential to improve the robustness and reliability of the predictive classifier through internal and external validation. Secondly, the classifier was developed using genus-level variables to ensure consistency in the results. However, employing metagenomic analysis could facilitate the identification of species-level biomarkers and provide additional insights into bacterial functions. Furthermore, this study provided an in-depth evaluation of the relationship between chemotherapy sensitivity and the oral microbiome, alongside an analysis of the compositional dynamics of the oral microbiota before and after neoadjuvant chemotherapy. Future research will aim to investigate the alterations and patterns of microbiota within TNBC tissue samples.

In conclusion, this study may provide evidence that oral microbiome profiling can be used to predict neoadjuvant chemotherapy sensitivity in patients with TNBC, by identifying taxonomic features characteristic of both pathogenic and commensal microbiome associated with differing treatment responses. Additionally, a significant correlation was observed between therapeutic outcomes and changes in microbial composition. These findings suggest that the oral microbiome holds potential as a predictive biomarker for assessing the responsiveness of TNBC patients to neoadjuvant chemotherapy. The predictive models established in this study may facilitate the integration of microbiome research into the therapeutic management of TNBC.

## Data Availability

The raw data supporting the conclusions of this article will be made available by the authors, without undue reservation.
